# A randomized trial of a digitally delivered, home-based neuromodulation and mindfulness intervention for pain management in older adults with knee osteoarthritis

**DOI:** 10.1038/s41746-026-02577-7

**Published:** 2026-03-31

**Authors:** Juyoung Park, Chiyoung Lee, Lifeng Lin, Mindy J. Fain, Ilknur Telkes, Stephen Dahmer, Lindsey Park, Jason Hoang, Hongyu Miao, Hyochol Ahn

**Affiliations:** 1 The University of Arizona College of Nursing, Tucson, AZ USA; 2 Department of Electrical & Computer Engineering, The University of Arizona College of Engineering, Tucson, AZ USA; 3 Department of Epidemiology and Biostatistics, The University of Arizona College of Public Health, Tucson, AZ USA; 4 Geriatrics and Palliative Medicine, The University of Arizona College of Medicine, Division of General Internal Medicine, Tucson, AZ USA; 5 University of Arizona Center on Aging, Tucson, AZ USA; 6 Department of Neurosurgery, The University of Arizona College of Medicine, Tucson, AZ USA; 7 Andrew Weil Center for Integrative Medicine, The University of Arizona, Tucson, AZ USA; 8 College of Nursing, Florida State University, Tallahassee, FL USA; 9 Department of Statistics, Florida State University, Tallahassee, FL USA; 10 Department of Computer Science, Florida State University, Tallahassee, FL USA

**Keywords:** Diseases, Health care, Medical research, Neuroscience

## Abstract

Knee osteoarthritis (OA) is a major cause of chronic pain and disability in older adults, yet scalable home-based interventions remain limited, partly due to the lack of clinically effective digital solutions. This study is the first fully powered randomized, double-blind, sham-controlled trial to test a digitally delivered, home-based protocol combining transcranial direct current stimulation (tDCS) and mindfulness-based meditation (MBM) for knee OA pain. A total of 208 participants were randomized to active tDCS + MBM, active tDCS + sham MBM, sham tDCS + active MBM, or double sham; they completed ten 20-min sessions over 2 weeks. Knee pain intensity was measured at baseline, post treatment, and monthly for 3 months. Although both active tDCS groups improved, the difference between the combined tDCS + MBM intervention and tDCS alone was not statistically significant. Benefits were not sustained at 3 months. These findings offer the first evidence that a remotely supervised, digitally delivered tDCS + MBM intervention can rapidly reduce knee OA pain, supporting future personalized and extended treatment studies. Clinical trial registration: ClinicalTrials.gov NCT04375072 (registered May 7, 2020).

## Introduction

Knee osteoarthritis (OA), characterized by joint degeneration, is one of the most common causes of chronic pain and disability in older adults. It significantly impairs mobility, reduces daily functioning, and negatively affects quality of life^1,2^. Conventional treatments, including pharmacological and joint-targeted procedures and systemic analgesics^3,4^, often provide only modest pain relief and carry risks of adverse effects, such as gastrointestinal issues and cardiovascular complications^5^. These limitations underscore the need for effective, scalable, and safe nonpharmacological intervention for knee OA pain management in aging populations^6^.

Transcranial direct current stimulation (tDCS) and mindfulness-based meditation (MBM) have each demonstrated clinically meaningful benefits in addressing both physiological and psychological dimensions of knee OA pain^7,8^. tDCS, a noninvasive, safe, and affordable digital stimulation technique^9^, applies low-intensity electrical currents to cortical regions involved in pain regulation, including the motor, somatosensory, and prefrontal cortices^10–12^. This stimulation modulates cortical excitability and enhances descending pain inhibition pathways^13–17^. MBM complements this neural approach by engaging attentional and emotional regulation pathways, which can reduce pain perception and improve coping strategies^18–20^. Meta-analyses have reported significant pain reductions with tDCS in persons with knee OA^21^ and mindfulness-based interventions have shown similar benefits in other chronic pain conditions^22^.

Combining interventions with distinct neural and behavioral mechanisms may yield additive or synergistic effects that exceed either modality alone^23–25^. Delivered together, these modalities may enhance knee pain relief by reinforcing neuroplastic changes and behavioral adaptations^26^. Early studies suggest feasibility^23^, but rigorous, fully powered evidence, particularly in home-based, digitally delivered formats, is lacking^24,25^. Given the growing emphasis on patient-centered, accessible care for older adults with mobility limitations, a digitally delivered, home-based protocol integrating tDCS and MBM may offer a scalable solution^23^. Most prior studies have been conducted in clinic settings, which can be burdensome for this population^27,28^. In contrast, remotely supervised home-based interventions can maintain treatment fidelity while enhancing access and adherence^23^.

Prior clinic-based studies have demonstrated that combining tDCS with mindfulness-based approaches is safe and effective for pain management. For example, McCallion et al.^29^ and Pimenta et al.^30^ reported improvements in pain and attention outcomes when mindfulness practices were paired with anodal tDCS in clinical settings. Building on this work, our group^23,31,32^ has demonstrated that home-based, remotely supervised tDCS, administered with or without concurrent MBM, is feasible, safe, and yields clinically meaningful reductions in knee OA pain comparable to in-clinic protocols, though in smaller samples. More specifically, these remotely delivered protocols achieved high session completion rates, no serious adverse events, and clinically meaningful reductions in pain intensity and osteoarthritis-related symptoms, with effect sizes comparable to or exceeding those reported in traditional in-clinic tDCS trials^33,34^.

This study contributes to the digital transformation of chronic knee OA pain care by evaluating a highly-deployable and home-based neuromodulation intervention that is remotely supervised and digitally supported. It represents the first fully powered, randomized, double-blind trial to assess a digitally delivered protocol integrating tDCS and MBM for chronic knee OA pain. This scalable, technology-enabled approach offers a promising alternative for individuals with mobility limitations. We conducted a randomized, double-blind, sham-controlled factorial trial comparing four groups: active tDCS + active MBM, active tDCS + sham MBM, sham tDCS + active MBM, and sham tDCS + sham MBM. The primary objective was to examine the combined analgesic effects (short-term and sustained) of tDCS and MBM on knee pain intensity. The secondary objective was to investigate whether the treatment effects were associated with covariates such as BMI, age, or sex.

## Results

**Fig. 1:**
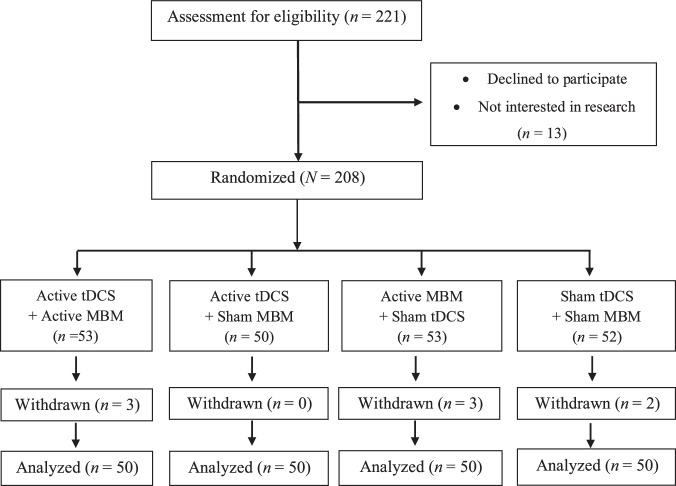
Participant flow through screening, randomization, and analysis. Flow diagram illustrating participant progression through the study. A total of 221 individuals were assessed for eligibility, of whom 208 were randomized. Participants were allocated to four groups: active transcranial direct current stimulation combined with active mindfulness-based meditation (tDCS+MBM; *n* = 53), active tDCS with sham MBM (*n* = 50), active MBM with sham tDCS (*n* = 53), and sham tDCS with sham MBM (*n* = 52). Withdrawals occurred in each group as indicated (*n* = 3, 0, 3, and 2, respectively), and 50 participants per group were included in the final analysis. MBM mindfulness-based meditation, tDCS transcranial direct current stimulation.

Of the 221 individuals screened, 13 declined participation and were excluded prior to randomization, resulting in a final sample of 208 participants analyzed under the ITT principle (Fig. 1). Participants were recruited and completed the 3-month follow-up data collection from August 2020 to January 2025. Of the 208 randomized participants, data from 8 were missing, and multiple outliers in the NRS outcome were retained in analyses to ensure conclusion robustness. Based on Shapiro-Wilk test and skewness calculation (e.g., for the NRS difference between Day 10 and baseline in the active tDCS + active MBM group, test statistic: 0.95; *p* value: 0.04; skewness: –0.68), the knee pain intensity outcome followed a skewed normal distribution rather than a symmetric one. No missing data were reported among the remaining 200 participants during the study follow-up. All participants completed every intervention session, demonstrating 100% adherence to the tDCS protocol. No adverse events were observed, and all participants tolerated the interventions well. No device- or study-related adverse events occurred.

Baseline demographic and clinical characteristics of the 208 randomized participants are summarized in Table 1. The mean age was 67.64 years; 68.1% were female and 82.6% were Caucasian. Groups were comparable in age, sex, race, marital status, and education (all *p* > 0.05).

**Table 1:** Demographic and baseline clinical characteristics of the participants^a^ (*N* = 208)

	mean (SD) or %				
	Active tDCS + Active MBM (*n* = 53)	Active tDCS + Sham MBM (*n* = 50)	Sham tDCS + Active MBM (*n* = 53)	Sham tDCS + Sham MBM (*n* = 52)	Total (*N* = 208)
**Age, years**	66.49 (7.74)	68.06 (7.42)	67.19 (7.89)	68.88 (6.92)	67.64 (7.51)
**Gender**					
female	34 (64.2%)	34 (68.0%)	40 (75.5%)	34 (65.4%)	142 (68.1%)
male	19 (35.8%)	16 (32.0%)	13 (24.5%)	18 (34.6%)	66 (31.7%)
**Race**	*Op*				
African American	7 (13.2%)	3 (6.0%)	4 (5.7%)	6 (11.5%)	20 (9.6%)
Asian	0 (0.0%)	0 (0.0%)	5 (9.4%)	1 (1.9%)	6 (2.9%)
Hispanic or Latino	2 (3.8%)	4 (8.0%)	3 (5.7%)	2 (3.8%)	11 (5.3%)
White	44 (83.0%)	43 (86.0%)	41 (77.4%)	43 (82.7%)	171 (82.6%)
**BMI, kg/m**2	31.83 (8.72)	29.03 (6.95)	31.40 (8.41)	31.16 (7.69)	30.85 (8.00)
**Marital Status**					
divorced	8 (15.1%)	9 (18.0%)	11 (20.8%)	12 (23.1%)	40 (19.2%)
living with partner	0 (0.0%)	2 (4.0%)	1 (1.9%)	1 (1.9%)	4 (1.9%)
married	35 (66.0%)	35 (70.0%)	28 (52.8%)	30 (57.7%)	128 (61.5%)
never married	6 (11.3%)	0 (0.0%)	7 (13.2%)	5 (9.6%)	18 (8.7%)
widowed	4 (7.5%)	4 (8.0%)	6 (11.3%)	4 (7.7%)	18 (8.7%)
**Education**					
< high school	2 (3.8%)	0 (0.0%)	0 (0.0%)	0 (0.0%)	2 (1.0%)
high school	9 (17.0%)	10 (20.0%)	10 (18.9%)	12 (23.1%)	41 (19.7%)
2-year college	10 (18.9%)	6 (12.0%)	11 (20.8%)	10 (19.2%)	37 (17.8%)
4-year college	21 (39.6%)	16 (32.0%)	17 (32.1%)	12 (23.1%)	66 (31.7%)
master’s degree	10 (18.9%)	11 (22.0%)	9 (17.0%)	10 (19.2%)	40 (19.2%)
doctoral degree	1 (1.9%)	7 (14.0%)	6 (11.3%)	8 (15.4%)	22 (10.6%)
**Average duration of osteoarthritis (Month)**	62.49 (59.01)	34.86 (39.97)	57.10 (69.63)	50.92 (58.58)	51.56 (58.44)
**Index knee**					
left	22 (41.5%)	26(52.0%)	28 (53.8%)	24 (46.2%)	100 (48.1%)
right	31 (58.5%)	24 (48.0%)	24 (46.2%)	28 (53.8%)	108 (51.9%)
**Kellgren-Lawrence score (index knee)**	2.91 (1.34)	2.86 (1.22)	2.96 (1.44)	2.80 (1.50)	2.88 (1.37)
**NRS**	42.40 (25.79)	44.94 (25.33)	41.67 (22.16)	42.13 (24.17)	42.80 (24.26)

*MBM* mindfulness-based meditation, *NRS* numeric rating scale, *SD* standard deviation, *tDCS* Transcranial Direct Current Stimulation.

^a^Kruskal-Wallis test was used for continuous variables and Chi-square test was used for discrete variables.

The mean BMI was 30.85 kg/m², consistent with Obesity Class I^35^ and reflecting known associations between obesity and knee OA^36,37^. The active tDCS + sham MBM group reported a shorter knee OA duration (34.86 ± 39.97 months) than other groups ( > 50 months), which indicated the necessity to include this variable in evaluation of intervention effects. Left- and right-knee OA were evenly distributed (48.1% vs. 51.9%). Baseline Kellgren-Lawrence scores (2.80–2.96) and NRS pain scores (mean = 42.80 ± 24.26) did not differ significantly across groups.

**Fig. 2:**
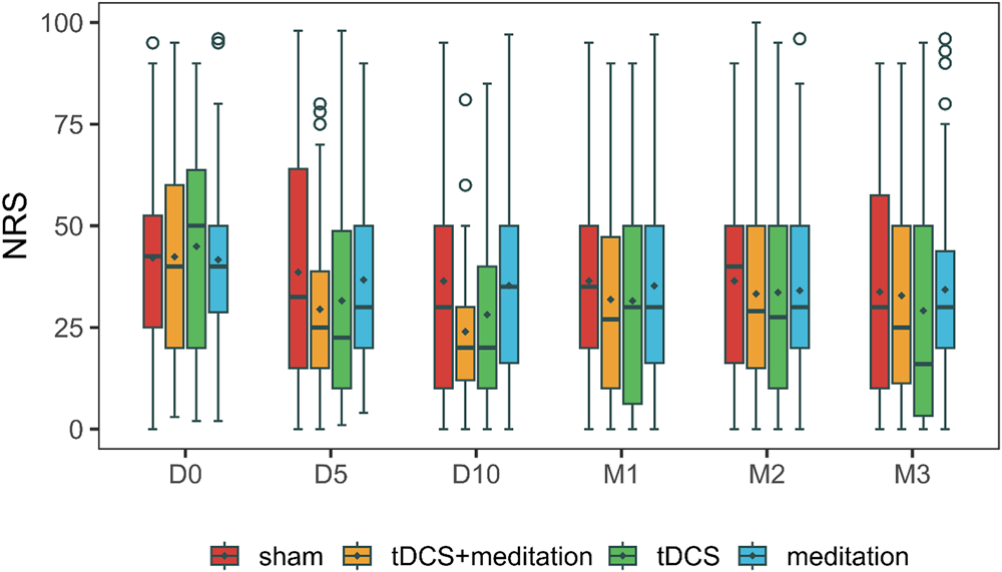
Pain intensity trajectories across intervention groups over time. Line plot showing Numeric Rating Scale (NRS) pain scores for all four groups across six time points: baseline, Day 5, Day 10, and Months 1–3 post-intervention. Each line represents the mean NRS score for a treatment group (sham, transcranial direct current stimulation [tDCS], meditation, and tDCS combined with meditation), with different colors corresponding to each group. Error bars represent standard errors of the mean.

Figure 2 presents NRS scores for all four groups across six time points. Baseline NRS distributions did not differ significantly across groups (Kruskal-Wallis *p* = 0.886; Table 1, Fig. 2). By Day 5, mean NRS scores decreased in all groups: active tDCS + active MBM ( − 30.5%), active tDCS + sham MBM ( − 29.8%), sham tDCS + active MBM ( − 11.9%), and sham MBM + sham tDCS ( − 8.4%). Reductions continued by Day 10: active tDCS + active MBM ( − 19.6 points; 43.5%), active tDCS + sham MBM ( − 16.0 points; 37.3%), sham tDCS + active MBM ( − 6.3 points; 15.2%), and sham tDCS + sham MBM ( − 5.8 points; 13%).

Compared to Day 10, NRS changes at Months 1–3 were sham tDCS + sham MBM (0.1%, 0.1%, –7.3%), active tDCS + active MBM (33.0%, 38.8%, 36.9%), active tDCS + sham MBM (11.9%, 19.5%, 3.4%), and sham tDCS + active MBM (–0.2%, –3.5%, –2.9%). Although the active tDCS + active MBM group and the active tDCS + sham MBM group had similar NRS means at Months 1, 2, and 3 post intervention, the percentage changes differed due to variations in pain levels at the end of treatment (Day 10).

Supplementary Figure 1 displays the trajectories of mean NRS scores over six time points for all four groups in this study. Knee pain in the active tDCS + active MBM group decreased most by Day 10, followed by a return toward baseline. In contrast, active tDCS + sham MBM showed moderate initial reduction by Day 10, gradual increase through Month 1 and Month 2, then the lowest score by Month 3. Sham tDCS + Active MBM exhibited a modest and stable decrease; the sham group showed minimal change.

For the primary analysis, an RLMM was used to compare changes in NRS scores from baseline to Day 10 across the four groups, adjusting for demographic and baseline clinical characteristics (Table 2). The goodness-of-fit metrics of RLMM used in the primary analysis were obtained, including a marginal *R*2 of 0.21, a conditional *R*2 of 0.75, an RMSE of 10.43, and a Sigma value of 13.07, all of which were numerically superior to those obtained using a regular LMM (i.e., a marginal *R*2 of 0.19, a conditional *R*2 of 0.73, an RMSE of 10.45, and a Sigma value of 13.75).

**Table 2:** RLMM analysis for comparing NRS measures between baseline and day 10, with demographic and baseline characteristics included^a^

	Time effect	*p* value	Group effect (between-group difference)	*p* value	Time × group interaction effect	*p* value
	Estimate [95% CI]		Estimate [95% CI]		Estimate [95% CI]	
Time: Baseline vs. Day 10	–6.68 [–12.16, –1.20]	0.018*				
**Study group**						
tDCS + MBM (vs. Sham)			–2.02 [–12.28, 8.24]	0.700	–12.05 [-19.88, –4.21]	0.003*
tDCS (vs. Sham)			4.01 [–5.82, 13.84]	0.425	–10.09 [-17.72, –2.46]	0.010*
MBM (vs. Sham)			–2.06 [–11.99, 7.88]	0.685	–0.33 [–8.07, 7.42]	0.935

tDCS + MBM refers to active tDCS combined with active MBM; tDCS refers to active tDCS with sham MBM; MBM refers to sham tDCS with active MBM; and sham refers to sham tDCS with sham MBM.

*RLMM* Robust Linear Mixed Model, *MBM* Mindfulness-Based Meditation, *NRS* Numeric Rating Scale, *tDCS* Transcranial Direct Current Stimulation.

*p <0.025 (Bonferroni-corrected significance level).

^a^The significance level after Bonferroni correction is α = 0.025.

Average duration of OA was not significantly associated with NRS outcomes (*p* = 0.973), although baseline duration differed across groups (Supplementary Table 1). Among all demographic and clinical variables examined, only BMI was significantly and positively associated with NRS measures (estimate: 0.69, 95% CI [0.26–1.12]; *p* = 0.002), indicating that higher BMI was linked to increased knee pain scores (Supplementary Table 1).

The main effect of group was not significant (*p* > 0.4), indicating no overall difference in average pain across groups. The main effect of time was significant (estimate -6.68, 95% CI [ − 12.16, −1.20]; *p* = 0.018), reflecting overall significant NRS reduction from baseline to Day 10 across all groups. By Day 10, the active tDCS + active MBM group exhibited a significant reduction in knee pain (estimate = –12.05, 95% CI [ − 19.88, −4.21]; *p* = 0.003; Cohen’s *d* ≈ 0.82), as did the active tDCS + sham MBM group (estimate = –10.09, 95% CI [ − 17.72, −2.46]; *p* = 0.010; Cohen’s *d* ≈ 0.67). Both groups demonstrated significant short-term reductions in pain relative to sham, whereas the sham tDCS + active MBM group showed a modest and nonsignificant change (estimate = –0.33, 95% CI [ − 8.07, 7.42]; *p* = 0.935; Table 2).

Between-group comparisons indicated no statistically significant difference between active tDCS alone and combined active tDCS + MBM for short-term pain reduction. The sham tDCS + sham MBM group showed a mean pain change of –5.8 points ( ~ 13%) by Day 10, likely reflecting placebo-related or natural symptom fluctuations.

A RLMM was used to assess changes in NRS scores from baseline through 3 months post intervention, adjusting for treatment group and baseline characteristics (Table 3). There was a significant main effect of time (estimate: –8.98, 95% CI [ − 16.08, −1.88]; *p* = 0.014), indicating an overall reduction in knee pain across all groups (Table 3). However, none of the active interventions showed a statistically significant difference from the sham group at 3 months: active tDCS + active MBM (estimate: –3.14, 95% CI [ − 13.92, 7.65]; *p* = 0.569), active tDCS + sham MBM (estimate: 4.87, 95% CI [ − 5.48, 15.22]; *p* = 0.357), and sham tDCS + active MBM (estimate: –3.21, 95% CI [ − 13.67, 7.25]; *p* = 0.548). These results suggest that, while knee pain improved over time, no intervention maintained long-term effects.

**Table 3:** RLMM analysis for comparing NRS measures between baseline and month 3, with demographic and baseline characteristics included^a^

	Time effect	*p* value	Group effect (between-group difference)	*p* value	Time × group interaction effect	*p* value
	Estimate [95% CI]		Estimate [95% CI]		Estimate [95% CI]	
Time: Baseline vs. Month 3	–8.98 [–16.08, –1.88]	0.014*				
**Study group**						
tDCS + MBM (vs. Sham)			–3.14 [–13.92, 7.65]	0.569	–2.13 [–12.28, 8.03]	0.682
tDCS (vs. Sham)			4.87 [–5.48, 15.22]	0.357	–7.53 [–17.42, 2.36]	0.137
MBM (vs. Sham)			–3.21 [–13.67, 7.25]	0.548	1.26 [–8.78, 11.30]	0.806

*Note*. tDCS + MBM refers to active tDCS combined with active MBM; tDCS refers to active tDCS with sham MBM; MBM refers to sham tDCS with active MBM; and sham refers to sham tDCS with sham MBM.

*RLMM* Robust Linear Mixed Model, *MBM* Mindfulness-Based Meditation, *NRS* Numeric Rating Scale, *tDCS* Transcranial Direct Current Stimulation.

*p <0.025 (Bonferroni-corrected significance level).

^a^The significance level after Bonferroni correction is α = 0.025.

At 3 months, group differences diminished. The active tDCS + active MBM group, despite the largest initial reduction, showed partial rebound and no longer differed significantly from sham groups. The active tDCS + sham MBM group showed continued improvement over time, yielding the lowest mean NRS score at 3 months. The active MBM + sham tDCS group demonstrated no sustained benefit beyond its initial modest effect. Among all demographic and clinical variables examined, only BMI was significantly and positively associated with NRS measures (estimate: 0.64, 95% CI [0.19, 1.09]; *p* = 0.005), indicating that higher BMI was linked to increased knee pain scores (Supplementary Table 2).

A RLMM model was used to accommodate NRS scores across all six time points to identify demographic and baseline predictors of pain response. BMI was the only significant predictor (estimate: 0.60, 95% CI [0.17, 1.03]; *p* = 0.0068). Sex-stratified effect sizes for intervention groups are presented descriptively (Supplementary Table 3).

**Fig. 3:**
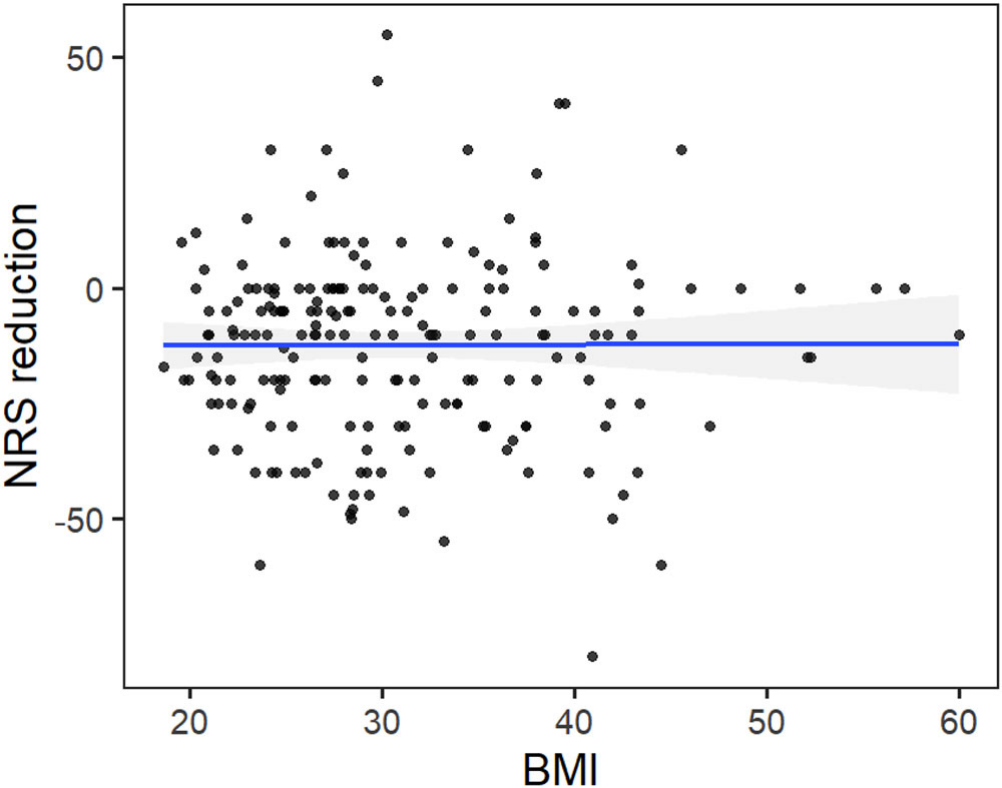
Association between body mass index and pain reduction across all participants. Scatter plot showing the relationship between body mass index (BMI) and change in pain intensity, calculated as the difference in Numeric Rating Scale (NRS) scores between Day 10 and Day 0 (NRS reduction = Day 10 − Day 0). Each point represents an individual participant. The solid blue line indicates the linear regression fit across all participants, and the shaded area represents the 95% confidence interval.

**Fig. 4:**
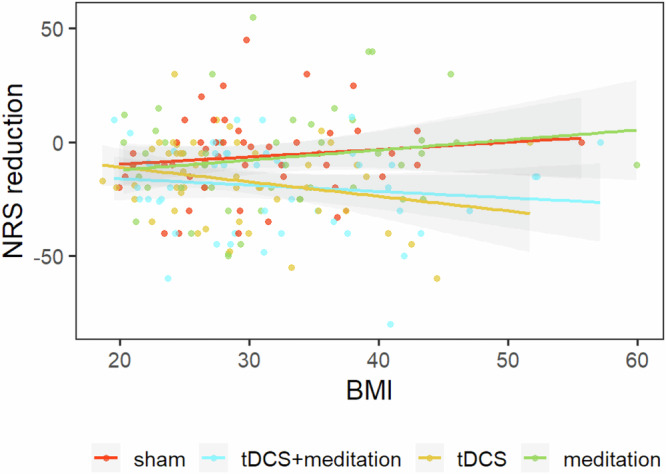
Association between body mass index and pain reduction across intervention groups. Scatter plot showing the relationship between body mass index (BMI) and change in pain intensity, measured as the difference in Numeric Rating Scale (NRS) scores between Day 10 and Day 0 (NRS reduction = Day 10 − Day 0). Each point represents an individual participant. Colored points and corresponding regression lines indicate treatment groups: sham (red), transcranial direct current stimulation combined with meditation (tDCS+meditation; blue), tDCS alone (yellow), and meditation alone (green). Solid lines represent linear regression fits, and shaded areas indicate95% confidence intervals.

Exploratory regression analyses examined whether BMI moderated knee pain reduction outcomes. Overall, BMI was not strongly associated with NRS knee pain reduction (Fig. 3); however, stratified analyses suggested that higher BMI was linked to greater benefit from the combined tDCS + MBM intervention for knee pain (Fig. 4).

## Discussion

This study represents the first fully powered, randomized, double-blind, sham-controlled trial to evaluate a digitally delivered, home-based intervention combining tDCS and MBM for chronic knee OA pain in older adults. The 2-week combined intervention produced a significant short-term reduction in knee pain immediately post-treatment; however, these effects declined over time, and group trajectories diverged across the 3-month follow-up. Throughout the section, “tDCS alone” and “MBM alone” refer to active tDCS + sham MBM and active MBM + sham tDCS, respectively.

Active tDCS produced significant short-term reductions in knee pain, both with and without concurrent MBM, whereas MBM alone did not demonstrate a statistically significant effect. Although the combined tDCS + MBM group exhibited numerically larger pain reductions, the difference between the combined intervention and tDCS alone was not statistically significant, indicating that the addition of MBM did not provide a clinically observable incremental analgesic benefit beyond tDCS in this trial^23,26^. While the combined intervention showed improvement relative to double sham, the present analyses do not demonstrate an additive or synergistic interaction effect of MBM when combined with tDCS. Importantly, this study was not powered to detect additive or synergistic interaction effects between modalities, and no statistically significant difference between tDCS + MBM and tDCS-only was observed. Taken together, these findings suggest that tDCS was the primary driver of short-term analgesic effects, while the contribution of MBM remains uncertain.

The observed short-term pain reductions in both active tDCS groups are consistent with prior home-based tDCS research, including a large randomized control trial reporting Cohen’s *d* = 1.2^38^, while meta-analyses of mindfulness-based interventions have generally reported smaller effects on chronic pain outcomes (Cohen’s *d* = 0.19–0.32)^39^. These findings reinforce the role of neuromodulation as an effective short-term, nonpharmacological approach for knee OA pain management in older adults.

Prior studies have suggested that concurrent neuromodulation and mindfulness engagement may optimize pain regulation through complementary neurophysiological and cognitive-affective mechanisms^26,40,41^. For example, Rodrigues et al.^40^ demonstrated an additive effect in anxiety treatment when D-cycloserine enhanced exposure therapy, and Pimenta et al.^26^ reported improvements in attention and headache-related disability when mindfulness was combined with anodal tDCS over the left dorsolateral prefrontal cortex in migraine patients. Additive effects have also been described in depression and migraine populations when tDCS is paired with cognitive or mindfulness-based therapies^26,41^ However, despite such prior evidence, the present trial did not demonstrate an additive or synergistic interaction effect of MBM when combined with tDCS. Accordingly, while prior literature supports the plausibility of synergistic mechanisms, confirmation of such effects will require trials specifically designed and powered to evaluate interaction effects using factorial designs or prespecified contrasts.

Sustained benefits of the intervention were limited. By one month, knee OA pain scores in the combined active group increased and no longer significantly differed from those in the sham groups, consistent with the time-limited effects of brief tDCS and behavioral protocols^42–44^. NRS scores increased by approximately 12–39% from Day 10 to the 1- or 2-month follow-up in both active tDCS+active MBM and active tDCS + sham MBM groups, suggesting that intervention effects lasted less than 1 month. These trajectories suggest that, while the combined intervention produced the greatest immediate knee OA pain relief, tDCS alone may yield more gradual but comparatively sustained benefits over time. At the primary endpoint, both active tDCS conditions produced clinically meaningful short-term reductions in knee OA pain^45^.

These findings suggest that, without ongoing maintenance, the effects of both tDCS and MBM diminish over time. tDCS-induced cortical excitability changes are transient, and meditation-related benefits may wane without continued practice. Sustaining gains in chronic knee OA pain may therefore require continued neuromodulation or psychological reinforcement beyond the initial treatment period. Evidence from prior studies suggests that extended or repeated tDCS protocols (e.g., 15–20 sessions) and continued MBM practice may enhance durability. Home-based booster sessions or ongoing MBM practice may offer flexible and scalable options for maintaining benefits.

In knee OA, reinforcing central inhibition and coping mechanisms is critical for achieving durable neuromodulatory and behavioral change^46–49^. Clinically, sustaining treatment benefits may require structured maintenance strategies, such as gradual tapering of stimulation sessions (e.g., three sessions in Week 3, two in Week 4, one in Week 5) or continued MBM practice supported by refresher resources or app-based tools^50^. Future studies should determine optimal dosing schedules and evaluate whether flexible maintenance protocols outperform fixed protocols in sustaining long-term pain relief^48^.

Prior literature has suggested that biological sex may influence responsiveness to neuromodulation and mind-body interventions targeting pain regulation^49,51^. However, the present study was not powered to formally test sex-by-treatment or sex-by-time interaction effects, and no statistical comparisons of treatment response by sex were conducted. Accordingly, any sex-stratified patterns suggested in prior work, or observed descriptively in this study, should be interpreted cautiously and viewed as exploratory rather than confirmatory. Future trials specifically designed and powered to evaluate sex-related neurobiological and psychosocial moderators may help inform more personalized multimodal pain management strategies.

Higher BMI was associated with greater knee pain intensity scores, likely reflecting increased joint loading, inflammation, and altered pain modulation^52^. Group-stratified analyses (Fig. 4) indicated that participants with higher BMI in the combined tDCS + MBM intervention group experienced greater reduction in knee pain, consistent with prior evidence that individuals with obesity may respond more strongly to neuromodulation due to elevated baseline symptom burden^53^. Higher BMI is often associated with central sensitization and impaired endogenous pain inhibition^54^, which may increase responsive to interventions targeting both neural and psychological pathways^26^. This finding supports the value of personalized approaches that considers BMI when optimizing multimodal pain interventions^55,56^. Despite variability in home settings, the observed effects underscore the ecological validity of the intervention and its real-world potential^57^. Future studies should explore whether tailored or booster protocols can help to sustain these benefits across BMI profiles.

Several limitations should be acknowledged. Although the study was designed to blind participants, neither treatment expectancy nor the success of participant blinding (e.g., post-intervention assessment of perceived treatment assignment) was formally assessed, which should be considered when interpreting the findings and may have implications for internal validity. With respect to intervention delivery, despite standardized protocols and real-time remote supervision, some variability in home-based tDCS administration may have occurred; however, devices were preprogrammed, electrode placement was verified, and staff were trained to minimize this risk. In terms of generalizability, participants were relatively well-educated older adults with access to technological support, and digital literacy may remain a barrier to broader implementation. Also, the MBM intervention was brief (2 weeks) and self-guided, without a live instructor component or formal fidelity assessment, which may have reduced the robustness of MBM effects compared with longer, instructor-led programs. Finally, although digital and CD options were provided, the requirement for access to playback devices and secure videoconferencing may have unintentionally excluded individuals with limited technology access.

Strengths of this trial include its large sample size, rigorous randomized double-blind sham-controlled design, and use of a fully powered analytic framework appropriate for longitudinal pain outcomes. The study is among the first to evaluate a remotely supervised, digitally delivered tDCS protocol at scale in older adults with knee OA, demonstrating high adherence, feasibility, and safety in real-world home settings. The inclusion of multiple control conditions allowed for a clear separation of tDCS-specific effects from placebo and nonspecific intervention effects. The use of robust linear mixed-effects modeling enhanced the reliability of findings in the presence of non-normal outcome distributions. Collectively, these strengths support the internal validity of the trial and underscore its contribution to advancing scalable, nonpharmacological pain management strategies for older adults with mobility limitations.

In conclusion, home-based tDCS produced clinically meaningful short-term reductions in knee OA pain, while MBM did not demonstrate independent or additive analgesic effects in this trial. Future studies should refine dosing, evaluate extended delivery and maintenance strategies, and employ trial designs powered to evaluate interaction effects in order to determine whether combined neuromodulation and mindfulness approaches can yield durable and personalized benefits.

## Methods

This study was approved by the Institutional Review Board at the University of Arizona (STUDY00003164) and registered at ClinicalTrials.gov (Identifier: NCT04375072; First posted: May 5, 2020). Written informed consent was obtained from all participants in accordance with the Declaration of Helsinki. The full trial protocol and statistical analysis plan can be accessed through the ClinicalTrials.gov study record (https://clinicaltrials.gov/study/NCT04375072). Recruitment occurred from August 2020 to January 2025. Participants completed follow-up assessments through 3 months post-intervention.

We conducted a double-blind, randomized, sham-controlled, Phase II parallel-group trial. A total of 208 participants with knee OA were randomized to one of four groups: active tDCS + active MBM (*n* = 53), active tDCS + sham MBM (*n* = 50), active MBM + sham tDCS (*n* = 53), or sham tDCS + sham MBM (*n* = 52). The participant flow diagram is shown in Fig. 1. Participants were randomized (1:1:1:1) using a pre-generated list created in R (version 4.4.3; R Foundation, Vienna, Austria) by the study statistician, balanced for age, race, and sex. Allocation was concealed in opaque envelopes.

Eligible participants were ages 50–85 and met American College of Rheumatology (ACR) clinical criteria for symptomatic knee OA^58^. Additional inclusion criteria were (a) knee OA pain within the past 3 months with an average Numeric Rating Scale (NRS) pain score ≥30 (0–100 scale), (b) English proficiency, and (c) no planned changes to pain medication during the trial. ACR clinical criteria required at least three of the following: (a) age >50, (b) morning stiffness <30 min, (c) crepitus, (d) bony tenderness, (e) bony enlargement, or (f) absence of palpable warmth. Key exclusion criteria were (a) history of brain surgery, tumor, seizure, stroke, epilepsy, or intracranial metal implants; (b) systemic rheumatic disorders (e.g., rheumatoid arthritis); (c) cognitive impairment (MMSE ≤ 23); (d) prosthetic knee replacement or nonarthroscopic knee surgery; or (e) no device access for secure video conferencing.

After the Institutional Review Board of the participating institution approved the research protocol, study participants were recruited using a multipronged community-based and clinic-based strategy. Recruitment methods included advertisements placed in local institutions and community locations (e.g., retail establishments, bus stops, buses, and local print and electronic media). Additional recruitment occurred through participation in community health fairs and educational programs organized by institutional and community partners, as well as through university-affiliated hospitals and local rheumatology clinics.

Active tDCS was delivered at 2 mA for 20 min per session, 5 days per week for 2 weeks. Participants self-administered stimulation using a 1 × 1 mini-CT Stimulator (Soterix Medical Inc., NY) with 5 × 7 cm saline-soaked sponge electrodes and single-position headgear to ensure consistent placement. The anode was placed over the primary motor cortex (C3 or C4) and the cathode was placed over the supraorbital region (Fp1 or Fp2), following established protocols^59^. Devices were preprogrammed with a one-time unlock code and automatically powered off after 20 min, per manufacturer protocol. Each session was digitally supervised in real time via secure videoconferencing. Study staff verified electrode placement, initiated device activation using a one-time code, and continuously monitored participants during the 20-min stimulation to ensure safety and adherence. Session attendance and completion were documented electronically. Sham tDCS used the same montage but delivered only a 30-s ramp-up and ramp-down to mimic sensations while delivering no active current beyond the initial period. This validated sham approach ensured that both participants and researchers remained blinded^25,33^.

Adherence to the study protocol was promoted throughout the trial. First, participants received baseline training until they demonstrated correct use of the tDCS device. Second, participants were remotely supervised by trained research staff at each stimulation to ensure proper technique; to monitor for adverse events; and to verify that participants safely operated the device, tolerated the session without adverse effects, and followed the study schedule. Third, the research team was trained using detailed procedural manuals on all aspects of the proposed research, including treatment protocols and participant interaction, in a step-by-step fashion. All personnel were trained before study initiation, and the principal investigator conducted weekly supervision to ensure protocol adherence.

MBM was delivered concurrently for 20 min per session over 2 weeks. Participants listened to a pre-recorded meditation CD guiding them through progressively deeper mindfulness practices, including controlled breathing, body awareness, and compassion. Participants accessed the guided meditation through a digital audio file compatible with computers, tablets, or smartphones; CDs and CD players were provided upon request. The same pre-recorded track was used across all sessions in either the MBM or sham MBM conditions to ensure consistency in content, duration, and structure. Sham MBM used identical equipment and format but excluded mindfulness content, instead instructing participants to relax and take deep breaths every 3 min. Deep breathing was incorporated in both conditions to control for relaxation and respiratory effects; however, only the active MBM intervention included sustained mindfulness and compassion-based practices intended to engage cognitive–affective pain modulation mechanisms. Both versions were standardized for posture, duration, and instruction^23^.

Participants, assessors, and analysts were blinded. The tDCS devices were identical and preprogrammed with built-in double-blinding features requiring a five-digit code to initiate stimulation. MBM and sham MBM were structurally matched, which means all participants were informed they would receive a meditation recording. Further, all staff were trained to minimize bias.

At baseline, demographic characteristics, including age (continuous), gender (male vs. female), race (African American, Asian, Hispanic or Latino, or White), body mass index (BMI; kg/m²), marital status (divorced, living with partner, married, never married, or widowed), and education ( < high school, high school, 2-year college, 4-year college, master’s degree, and doctoral degree), as well as clinical characteristics, such as the average duration of OA (months), index knee (the most affected knee), and Kellgren-Lawrence score (the severity of radiographic OA)^60^ were collected.

Pain intensity was primarily assessed using the NRS, where participants rated average knee OA pain over the past 24 h on a scale of 0 (no pain) to 100 (worst pain imaginable)^61^. The NRS is a validated, reliable tool (Cronbach’s α ≥ 0.8) widely used in knee OA studies and is suitable for older adults due to its simplicity^62,63^. Ratings were collected at six time points: Day 0 (baseline), Day 5 (mid-treatment), Day 10 (end of treatment), and at 1, 2, and 3 months post intervention.

This double-blind, randomized, sham-controlled Phase II trial used a 2 × 2 factorial design, with participants assigned to one of four groups at equal probability: (a) active tDCS + active MBM, (b) active tDCS + sham MBM, (c) sham tDCS + active MBM, or (d) sham tDCS + sham MBM. The target enrollment of 200 participants (assuming 10% attrition) yielded an effective sample size of 180, providing > 80% power to detect a ≥ 2-point reduction in NRS knee pain, which is considered clinically meaningful for chronic knee pain. Based on prior home-based tDCS and MBM data (Cohen’s *d* = 0.8–1.8), a conservative effect size of 0.4 and a Bonferroni-adjusted α = .025 were assumed (two tests: baseline vs. Day 10, and Day 10 vs. follow-up). Power estimation used a robust linear mixed-effects model that accounted for within-subject correlations across repeated measures (baseline, Days 5 and 10, and Months 1–3). With anticipated >90% retention, the design ensured adequate power to detect intervention efficacy under the intention-to-treat framework.

Raw data were manually reviewed for quality checking; outliers and missing values were retained to preserve variability and avoid introducing bias. Descriptive statistics summarized baseline characteristics. The normality of NRS knee pain scores was assessed using the Shapiro-Wilk test. A robust linear mixed-effects model (RLMM)^64^ was employed to accommodate the skewed distributions of outcomes and evaluate intervention effects at post intervention (Day 10) and across the full follow-up period (baseline, Days 5 and 10, and Months 1–3), adjusting for covariates, including BMI. The primary endpoint was NRS knee pain scores at Day 10 (post-intervention), and the secondary endpoints were NRS knee pain scores at 1-, 2-, and 3-month follow-up visits to assess sustained effects. Sustained effects and potential demographic or baseline predictors (e.g., age, sex, BMI) were examined within the same modeling framework. A Bonferroni-adjusted significance level of α = 0.025 was applied. All statistical analyses were conducted using R. This double-blind, randomized, sham-controlled, Phase II parallel-group trial focused on efficacy rather than feasibility outcomes. Adherence was tracked through session attendance logs during each remotely supervised session, and any missed sessions were recorded. Missing data were handled using an intention-to-treat approach to ensure robust conclusions.

## Supplementary information

Supplementary Information

## Data Availability

All data supporting the findings of this study are summarized in this published article. Requests for additional de-identified data will be reviewed by the corresponding author on a case-by-case basis and may require institutional and ethical approval.

## Code availability

No custom code was used in this study.

## References

[1] Liampas I. et al. The contribution of functional near-infrared spectroscopy (fNIRS) to the study ofneurodegenerative disorders: A narrative review. Diagnostics (Basel) **14**, 663 (2024). 10.3390/diagnostics14060663 PMC10969335 38535081

[2] Gelber, A. C. Knee osteoarthritis. *Ann. Intern Med.* **177**, Itc129–itc144 (2024). Sep. 39250809 10.7326/ANNALS-24-01249

[3] Wang, D., Chai, X.-Q., Hu, S.-S. & Pan, F. Joint synovial macrophages as a potential target for intra-articular treatment of osteoarthritis-related pain. *Osteoarthr. Cartil.* **30**, 406–415 (2022). 10.1016/j.joca.2021.11.014 34861384

[4] Stewart, M., Cibere, J., Sayre, E. C. & Kopec, J. A. Efficacy of commonly prescribed analgesics in the management of osteoarthritis: A systematic review and meta-analysis. *Rheumatol. Int.* **38**, 1985–1997 (2018). 30120508 10.1007/s00296-018-4132-z

[5] Wilcox, Jr. C. M. Gastrointestinal considerations in patients with cardiovascular disease using nonopioid analgesics for mild-to-moderate pain or cardioprotection. *Am. J. Cardiol.* **97**, 17–22 (2006). 16675318 10.1016/j.amjcard.2006.02.019

[6] Chopra, S., Kodali, R. T., McHugh, G. A., Conaghan, P. G. & Kingsbury, S. R. Home-based health care interventions for people aged 75 years and above with chronic, noninflammatory musculoskeletal pain: A scoping review. *J. Geriatr. Phys. Ther.* **46**, 3–14 (2023). 36525074 10.1519/JPT.0000000000000334

[7] Ahn, H. et al. Feasibility and efficacy of remotely supervised cranial electrical stimulation for pain in older adults with knee osteoarthritis: A randomized controlled pilot study. *J. Clin. Neurosci.* **77**, 128–133 (2020). 32402609 10.1016/j.jocn.2020.05.003 PMC7308202

[8] Badran, B. W. et al. A double-blind study exploring the use of transcranial direct current stimulation (tDCS) to potentially enhance mindfulness meditation (E-Meditation). *Brain Stimul.* **10**, 152–154 (2017). 27839723 10.1016/j.brs.2016.09.009

[9] Brunoni, A. R. et al. Digitalized transcranial electrical stimulation: A consensus statement. *Clin. Neurophysiol.* **143**, 154–165 (2022). 2022/11/01/. 36115809 10.1016/j.clinph.2022.08.018 PMC10031774

[10] Harms, A., Heredia-Rizo, A. M., Moseley, G. L., Hau, R. & Stanton, T. R. A feasibility study of brain-targeted treatment for people with painful knee osteoarthritis in tertiary care. *Physiother. Theory Pract.* **36**, 142–156 (2020). 29889597 10.1080/09593985.2018.1482391

[11] Moshfeghinia, R. et al. The effects of transcranial direct-current stimulation (tDCS) on pain intensity of patients with fibromyalgia: A systematic review and meta-analysis. *BMC Neurol.* **23**, 395 (2023). 37919664 10.1186/s12883-023-03445-7 PMC10621179

[12] Caumo, W. et al. Efficacy of home-based transcranial direct current stimulation over the primary motor cortex and dorsolateral prefrontal cortex in the disability due to pain in fibromyalgia: A factorial sham-randomized clinical study. *J. pain.* **25**, 376–392 (2024). 37689323 10.1016/j.jpain.2023.09.001

[13] Teixeira, P. E. P. et al. The analgesic effect of transcranial direct current stimulation (tDCS) combined with physical therapy on common musculoskeletal conditions: A systematic review and meta-analysis. *Princ. Pract. Clin. Res* **6**, 23–26 (2020). 32766451 10.21801/ppcrj.2020.61.5 PMC7406123

[14] Yao, J. et al. Analgesia induced by anodal tDCS and high-frequency tRNS over the motor cortex: Immediate and sustained effects on pain perception. *Brain Stimul.* **14**, 1174–1183 (2021). 34371209 10.1016/j.brs.2021.07.011

[15] Ma, Q. A functional subdivision within the somatosensory system and its implications for pain research. *Neuron* **110**, 749–769 (2022). 35016037 10.1016/j.neuron.2021.12.015 PMC8897275

[16] Giannoni-Luza, S. et al. Noninvasive motor cortex stimulation effects on quantitative sensory testing in healthy and chronic pain subjects: A systematic review and meta-analysis. *Pain* **161**, 1955–1975 (2020). 32453135 10.1097/j.pain.0000000000001893 PMC7679288

[17] Niddam, D. M., Wang, S.-J. & Tsai, S.-Y. Pain sensitivity and the primary sensorimotor cortices: A multimodal neuroimaging study. *Pain* **162**, 846–855 (2021). 32947544 10.1097/j.pain.0000000000002074

[18] Zeidan, F., Grant, J., Brown, C., McHaffie, J. & Coghill, R. Mindfulness meditation-related pain relief: Evidence for unique brain mechanisms in the regulation of pain. *Neurosci. Lett.* **520**, 165–173 (2012). 22487846 10.1016/j.neulet.2012.03.082 PMC3580050

[19] Zeidan, F. et al. Mindfulness meditation-based pain relief employs different neural mechanisms than placebo and sham mindfulness meditation-induced analgesia. *J. Neurosci.* **35**, 15307–15325 (2015). 26586819 10.1523/JNEUROSCI.2542-15.2015 PMC4649004

[20] Zeidan, F. et al. Brain mechanisms supporting the modulation of pain by mindfulness meditation. *J. Neurosci.* **31**, 5540–5548 (2011). 21471390 10.1523/JNEUROSCI.5791-10.2011 PMC3090218

[21] Yang, J. M. et al. Transcranial direct current stimulation for knee osteoarthritis: A systematic review and meta-analysis of randomized controlled. *Trials Arthritis Care Res.* **76**, 376–384 (2024). 10.1002/acr.25249 37779486

[22] Majeed, M. H., Ali, A. A. & Sudak, D. M. Mindfulness-based interventions for chronic pain: Evidence and applications. *Asian J. Psychiatry* **32**, 79–83 (2018). 10.1016/j.ajp.2017.11.025 29220782

[23] Ahn, H. et al. Efficacy of combining home-based transcranial direct current stimulation with mindfulness-based meditation for pain in older adults with knee osteoarthritis: A randomized controlled pilot study. *J. Clin. Neurosci.* **70**, 140–145 (2019). 31421990 10.1016/j.jocn.2019.08.047

[24] Ramasawmy, P. et al. No add-on therapeutic benefit of at-home anodal tDCS of the primary motor cortex to mindfulness meditation in patients with fibromyalgia. *Clin. Neurophysiol.* **164**, 168–179 (2024). 38901112 10.1016/j.clinph.2024.05.018

[25] Ahn, H. et al. Efficacy of transcranial direct current stimulation over primary motor cortex (anode) and contralateral supraorbital area (cathode) on clinical pain severity and mobility performance in persons with knee osteoarthritis: An experimenter- and participant-blinded, randomized, sham-controlled pilot clinical study. *Brain Stimul.* **10**, 902–909 (2017). 28566193 10.1016/j.brs.2017.05.007 PMC5568498

[26] Pimenta, L. D. S. et al. Effects of synergism of mindfulness practice associated with transcranial direct-current stimulation in chronic migraine: Pilot, randomized, controlled, double-blind clinical trial. *Front. Hum. Neurosci.* **15**, 769619 (2021). 34955789 10.3389/fnhum.2021.769619 PMC8692277

[27] Ramasawmy, P., Khalid, S., Petzke, F. & Antal, A. Pain reduction in fibromyalgia syndrome through pairing transcranial direct current stimulation and mindfulness meditation: A randomized, double-blinded, sham-controlled pilot clinical trial. *Front Med (Lausanne)* **9**, 908133 (2022). 36314032 10.3389/fmed.2022.908133 PMC9596988

[28] Hunter, M. A. et al. Mindfulness-based training with transcranial direct current stimulation modulates neuronal resource allocation in working memory: A randomized pilot study with a nonequivalent control group. *Heliyon* **4**, e00685 (2018). 30094362 10.1016/j.heliyon.2018.e00685 PMC6077241

[29] McCallion, E., Robinson, C. S., Clark, V. P. & Witkiewitz, K. Efficacy of transcranial direct current stimulation-enhanced mindfulness-based program for chronic pain: a single-blind randomized sham controlled pilot study. *Mindfulness* **11**, 895–904 (2020).

[30] Alamam, D. M. et al. Low back pain–related disability is associated with pain-related beliefs across divergent non–English-speaking populations: systematic review and meta-analysis. *Pain. Med.* **22**, 2974–2989 (2021). 33624814 10.1093/pm/pnaa430

[31] Lee, C. et al. Home-based, remotely supervised transcranial direct current stimulation improves the overall pain experience of older adults with knee osteoarthritis. *Pain. Res. Manag.* **2025**, 1783171 (2025). 40040747 10.1155/prm/1783171 PMC11876529

[32] Park, J., Lee, C., Fain, M., Lin, L. & Ahn, H. Home-based transcranial direct current stimulation improves central pain mechanisms and clinical pain in knee osteoarthritis: A randomized controlled trial. *Osteoarthr. Cartil.* **33**, S483–S484 (2025).

[33] Harvey, M.-P. et al. Relieving chronic musculoskeletal pain in older adults using transcranial direct current stimulation: Effects on pain intensity, quality, and pain-related outcomes. *Front. Pain. Res.* **3**, 817984 (2022). 10.3389/fpain.2022.817984 PMC9069524 35529592

[34] Antonioni, A., Baroni, A., Fregna, G., Ahmed, I. & Straudi, S. The effectiveness of home-based transcranial direct current stimulation on chronic pain: A systematic review and meta-analysis. *Digital Health* **10**, 20552076241292677 (2024). 39600390 10.1177/20552076241292677 PMC11590159

[35] Weir C. B., Jan A. BMI Classification Percentile And Cut Off Points. [Updated 2023 Jun 26]. In: StatPearls [Internet]. Treasure Island (FL): StatPearls Publishing. Accessed January 19, 2025. https://www.ncbi.nlm.nih.gov/books/NBK541070/.

[36] Marks, R. Obesity profiles with knee osteoarthritis: correlation with pain, disability, disease progression. *Obes. (Silver Spring)* **15**, 1867–1874 (2007). 10.1038/oby.2007.221 17636106

[37] Rogers, M. W. & Wilder, F. V. The association of BMI and knee pain among persons with radiographic knee osteoarthritis: A cross-sectional study. *BMC Musculoskelet. Disord.* **9**, 163 (2008). 19077272 10.1186/1471-2474-9-163 PMC2651875

[38] Martorella, G. et al. Self-administered transcranial direct current stimulation for pain in older adults with knee osteoarthritis: A randomized controlled study. *Brain Stimul.* **15**, 902–909 (2022). 35690388 10.1016/j.brs.2022.06.003 PMC9387776

[39] Hilton, L. et al. Mindfulness meditation for chronic pain: Systematic review and meta-analysis. *Ann. Behav. Med* **51**, 199–213 (2017). 27658913 10.1007/s12160-016-9844-2 PMC5368208

[40] Rodrigues, H. et al. Does D-cycloserine enhance exposure therapy for anxiety disorders in humans? A meta-analysis. *PLoS One* **9**, e93519 (2014). 24991926 10.1371/journal.pone.0093519 PMC4081005

[41] Aust, S. et al. Efficacy of augmentation of cognitive behavioral therapy with transcranial direct current stimulation for depression: A randomized clinical trial. *JAMA psychiatry* **79**, 528–537 (2022). 35442431 10.1001/jamapsychiatry.2022.0696 PMC9021985

[42] Ngernyam, N. et al. The effects of transcranial direct current stimulation in patients with neuropathic pain from spinal cord injury. *Clin. Neurophysiol.* **126**, 382–390 (2015). 25027640 10.1016/j.clinph.2014.05.034

[43] Stagg, C. J. & Nitsche, M. A. Physiological basis of transcranial direct current stimulation. *Neuroscientist* **17**, 37–53 (2011). 21343407 10.1177/1073858410386614

[44] O’Connell N. E., Marston L., Spencer S., DeSouza L. H., Wand B. M. Non-invasive brain stimulation techniques forchronic pain. Cochrane Database of Systematic Reviews. CD008208 10.1002/14651858.CD008208.pub4 (2018). 10.1002/14651858.CD008208.pub4 PMC7039253 29547226

[45] Wu, Y. L. et al. Effects of transcranial direct current stimulation on pain and physical function in patients with knee osteoarthritis: A systematic review and meta-analysis. *BMC Musculoskelet. Disord.* **25**, 703 (2024). 39227806 10.1186/s12891-024-07805-3 PMC11370230

[46] Arendt-Nielsen, L. et al. Assessment and manifestation of central sensitisation across different chronic pain conditions. *Eur. J. Pain.* **22**, 216–241 (2018). 29105941 10.1002/ejp.1140

[47] Zhou, D. et al. Transcranial direct current stimulation combined with repetitive transcranial magnetic stimulation for depression: A randomized clinical trial. *JAMA Netw. Open* **7**, e2444306–e2444306 (2024). 39535797 10.1001/jamanetworkopen.2024.44306 PMC11561687

[48] Monnart, A. et al. Treatment of resistant depression: A pilot study assessing the efficacy of a tDCS-mindfulness program compared with a tDCS-relaxation program. *Front. psychiatry* **10**, 730 (2019). 31708808 10.3389/fpsyt.2019.00730 PMC6819945

[49] McDonnell C. *Sex differences in pain sensitivity, pain modulation, and predictors of pain intensity in patients with opioid-treated chronic lower back pain*. Boston University; (2021).

[50] Ruffini, G. et al. Multichannel tDCS with advanced targeting for major depressive disorder: A tele-supervised at-home pilot study. *Front. Psychiatry* **15**, 1427365 (2024). 39211540 10.3389/fpsyt.2024.1427365 PMC11358063

[51] JJayathilake N. J., Phan T. T., Kim J., Lee K. P., Park J. M. Modulating neuroplasticity for chronic pain relief: Noninvasiveneuromodulation as a promising approach. *Exp. Molecul. Med.* **57**,1–14 (2025). 10.1038/s12276-025-01409-0 PMC11958754 40025172

[52] Chin, S.-H., Huang, W.-L., Akter, S. & Binks, M. Obesity and pain: A systematic review. *Int. J. Obes.* **44**, 969–979 (2020). 10.1038/s41366-019-0505-y 31848456

[53] Park, J. et al. Comparison of responders and nonresponders with knee osteoarthritis after transcranial direct current stimulation. *Pain. Manag.* **14**, 507–518 (2024). 39548963 10.1080/17581869.2024.2429943 PMC11721761

[54] Okifuji A., Hare B. D. The association between chronic pain and obesity. *J. Pain Res*. 399-408 (2015). 10.2147/JPR.S55598 PMC4508090 26203274

[55] Vincent, H. K., George, S. Z., Seay, A. N., Vincent, K. R. & Hurley, R. W. Resistance exercise, disability, and pain catastrophizing in obese adults with back pain. *Med. Sci. sports Exerc.* **46**, 1693 (2014). 25133997 10.1249/MSS.0000000000000294 PMC4137474

[56] Mathew J. *Neurofeedback training for pain management in people with knee osteoarthritis*. University of Otago; (2022).

[57] Hsieh, Y. -w et al. Effects of home-based versus clinic-based rehabilitation combining mirror therapy and task-specific training for patients with stroke: A randomized crossover trial. *Arch. Phys. Med. Rehab.* **99**, 2399–2407 (2018). 10.1016/j.apmr.2018.03.017 29702070

[58] Altman, R. et al. Development of criteria for the classification and reporting of osteoarthritis. Classification of osteoarthritis of the knee. *Diagn. Ther. Criteria Comm. Am. Rheum. Assoc. Arthritis Rheum.* **29**, 1039–1049 (1986). 10.1002/art.1780290816 3741515

[59] Charvet, L. E. et al. Remotely-supervised transcranial direct current stimulation (tDCS) for clinical trials: guidelines for technology and protocols. *Front Syst. Neurosci.* **9**, 26 (2015). 25852494 10.3389/fnsys.2015.00026 PMC4362220

[60] Kellgren, J. H. & Lawrence, J. Radiological assessment of osteo-arthrosis. *Ann. Rheum. Dis.* **16**, 494–502 (1957). 13498604 10.1136/ard.16.4.494 PMC1006995

[61] Fraenkel, L. et al. Measuring pain impact versus pain severity using a numeric rating scale. *J. Gen. Intern. Med.* **27**, 555–560 (2012). 22081365 10.1007/s11606-011-1926-z PMC3326111

[62] Alghadir, A. H., Anwer, S., Iqbal, A. & Iqbal, Z. A. Test-retest reliability, validity, and minimum detectable change of visual analog, numerical rating, and verbal rating scales for measurement of osteoarthritic knee pain. *J. Pain. Res*. **11**, 851–856 (2018). 29731662 10.2147/JPR.S158847 PMC5927184

[63] Ornetti, P., Dougados, M., Paternotte, S., Logeart, I. & Gossec, L. Validation of a numerical rating scale to assess functional impairment in hip and knee osteoarthritis: comparison with the WOMAC function scale. *Ann. Rheum. Dis.* **70**, 740–746 (2011). 21149497 10.1136/ard.2010.135483

[64] Koller, M. Robustlmm: An R package for robust estimation of linear mixed-effects models. *J. Stat. Softw.* **75**, 1–24 (2016).32655332

