# Supplementary material for: A randomized trial of a digitally delivered, home-based neuromodulation and mindfulness intervention for pain management in older adults with knee osteoarthritis
Source: NPJ Digit Med. 2026 Mar 31;9:397. doi: 10.1038/s41746-026-02577-7 (PMC13199477; doi:10.1038/s41746-026-02577-7)
Supplement: Supplementary file 1 — Supplementary Information [file 41746_2026_2577_MOESM1_ESM.pdf]

Supplementary Table 1. RLMM Analysis for Comparing NRS Measures Between Baseline and Day 10, With Demographic and Baseline Characteristics Included[a]

Variables	Estimate (95% CI)	t score	*p* value
(Intercept)	48.50 (-6.85 to 103.85)	1.72	0.088
Time: Baseline vs. Month 3	-6.68 (-12.16 to -1.20)	-2.39	0.018*
tDCS + MBM (vs. Sham)	-2.02 (-12.28 to 8.24)	-0.39	0.700
tDCS (vs. Sham)	4.01 (-5.82 to 13.84)	0.80	0.425
MBM (vs. Sham)	-2.06 (-11.99 to 7.88)	-0.41	0.685
Age	-0.18 (-0.67 to 0.31)	-0.72	0.473
Female (vs. Male)	7.93 (0.66 to 15.20)	2.14	0.034
Black (vs. Asian)	-7.71 (-33.13 to 17.71)	-0.59	0.553
White (vs. Asian)	-17.85 (-40.59 to 4.89)	-1.54	0.126
Hispanic (vs. Asian)	-5.48 (-32.08 to 21.12)	-0.40	0.687
BMI	0.69 (0.26 to 1.12)	3.09	0.002*
Widowed (vs. Married)	4.96 (-7.94 to 17.86)	0.75	0.453
Divorced (vs. Married)	4.63 (-3.92 to 13.18)	1.06	0.290
Never married (vs. Married)	2.43 (-10.19 to 15.05)	0.38	0.706
Living with partner (vs. Married)	-0.65 (-24.19 to 22.89)	-0.05	0.957
High school (vs. < High school)	0.63 (-31.57 to 32.83)	0.04	0.970
2-year college (vs. < High school)	-4.46 (-36.56 to 27.64)	-0.27	0.786
4-year college (vs. < High school)	-3.39 (-35.24 to 28.46)	-0.21	0.835
Master’s degree (vs. < High school)	-4.82 (-37.26 to 27.62)	-0.29	0.771
Doctoral degree (vs. < High school)	-8.56 (-41.94 to 24.82)	-0.50	0.616
Average duration of osteoarthritis (months)	-0.00 (-0.06 to 0.06)	-0.03	0.973
Index knee: Right vs. Left	2.13 (-4.40 to 8.66)	0.64	0.524
Kellgren-Lawrence score (index knee)	-1.34 (-3.87 to 1.19)	-1.04	0.300
Time × (tDCS + MBM vs. Sham)	-12.05 (-19.89 to -4.21)	-3.01	0.003*
Time × (tDCS vs. Sham)	-10.09 (-17.72 to -2.46)	-2.59	0.010*
Time × (MBM vs. Sham)	-0.33 (-8.07 to 7.42)	-0.08	0.935

[a]The significance level after Bonferroni correction is $\alpha = 0.025$.

Abbreviations: BMI = Body Mass Index, CI = Confidence Intervals, LMM = Linear Mixed Model, MBM = Mindfulness-Based Meditation,
NRS = Numeric Rating Scale, tDCS = Transcranial Direct Current Stimulation
tDCS + MBM refers to active tDCS combined with active MBM; tDCS refers to active tDCS with sham MBM; MBM refers to sham tDCS with active MBM; and sham refers to sham tDCS with sham MBM

Supplementary Table 2. RLMM Analysis for Comparing NRS Measures Between Baseline and 3 Months Post Intervention, With Demographic and Baseline Characteristics Included[a]

Variables	Estimate (95% CI)	t score	*p* value
(Intercept)	58.40 (2.80 to 114.00)	2.06	0.041
Time: Baseline vs. Month 3	-8.98 (-16.08 to -1.88)	-2.48	0.014*
tDCS + MBM (vs. Sham)	-3.14 (-13.92 to 7.65)	-0.57	0.569
tDCS (vs. Sham)	4.87 (-5.48 to 15.22)	0.92	0.357
MBM (vs. Sham)	-3.21 (-13.67 to 7.25)	-0.60	0.548
Age	-0.16 (-0.65 to 0.33)	-0.62	0.537
Female (vs. Male)	7.61 (0.32 to 14.90)	2.05	0.042
Black (vs. Asian)	-8.02 (-33.54 to 17.50)	-0.62	0.539
White (vs. Asian)	-21.13 (-43.94 to 1.68)	-1.81	0.071
Hispanic (vs. Asian)	-12.94 (-39.64 to 13.76)	-0.95	0.344
BMI	0.64 (0.19 to 1.09)	2.84	0.005*
Widowed (vs. Married)	4.00 (-8.96 to 16.96)	0.61	0.546
Divorced (vs. Married)	3.88 (-4.70 to 12.46)	0.89	0.376
Never married (vs. Married)	4.83 (-7.83 to 17.49)	0.75	0.456
Living with partner (vs. Married)	-5.28 (-28.90 to 18.34)	-0.44	0.662
High school (vs. < High school)	-6.34 (-38.66 to 25.98)	-0.38	0.701
2-year college (vs. < High school)	-11.80 (-44.02 to 20.42)	-0.72	0.474
4-year college (vs. < High school)	-9.89 (-41.86 to 22.08)	-0.61	0.545
Master's degree (vs. < High school)	-10.03 (-42.61 to 22.55)	-0.60	0.547
Doctoral degree (vs. < High school)	-14.44 (-47.96 to 19.08)	-0.84	0.400
Average duration of osteoarthritis (months)	0.01 (-0.05 to 0.07)	0.32	0.748
Index knee: Right vs. Left	2.56 (-4.01 to 9.13)	0.76	0.446
Kellgren-Lawrence score (index knee)	-1.54 (-4.09 to 1.01)	-1.19	0.236
Time × (tDCS + MBM vs. Sham)	-2.13 (-12.28 to 8.03)	-0.41	0.682
Time × (tDCS vs. Sham)	-7.53 (-17.42 to 2.36)	-1.49	0.137
Time × (MBM vs. Sham)	1.26 (-8.78 to 11.30)	0.25	0.806

[a]The significance level after Bonferroni correction is $\alpha = 0.025$.

Abbreviations: BMI = Body Mass Index, CI = Confidence Intervals, LMM = Linear Mixed Model, MBM = Mindfulness-Based Meditation,

NRS = Numeric Rating Scale, tDCS = Transcranial Direct Current Stimulation

tDCS + MBM refers to active tDCS combined with active MBM; tDCS refers to active tDCS with sham MBM; MBM refers to sham tDCS with active MBM; and sham refers to sham tDCS with sham MBM.

Supplementary Table 3. Effect Sizes of Interventions for Males and Females in Terms of Cohen's d, With 95% Confidence Intervals in Brackets

	Sex	Day 10 vs. baseline	Baseline vs. Month 3
sham tDCS + sham MBM	male	0.34 [0.09, 0.58]	0.57 [0.30, 0.84]
	female	0.19 [-0.06, 0.45]	0.25 [-0.14, 0.64]
active tDCS + active MBM	male	0.40 [0.13, 0.68]	0.07 [-0.42, 0.57]
	female	1.08 [0.67, 1.49]	0.59 [0.20, 0.98]
active tDCS + sham MBM	male	0.59 [0.23, 0.94]	0.46 [0.01, 0.91]
	female	0.72 [0.42, 1.01]	0.60 [0.23, 0.97]
sham tDCS + active MBM	male	0.14 [-0.23, 0.51]	0.17 [-0.22, 0.56]
	female	0.41 [0.01, 0.80]	0.44 [-0.00, 0.89]

Abbreviations: MBM = Mindfulness-Based Meditation; tDCS = Transcranial Direct Current Stimulation
tDCS + MBM refers to active tDCS combined with active MBM; tDCS refers to active tDCS with sham MBM; MBM refers to sham tDCS with active MBM; and sham refers to sham tDCS with sham MBM.

Supplementary Figure 1. Trajectories of mean NRS scores over six time points for all four groups in this study.

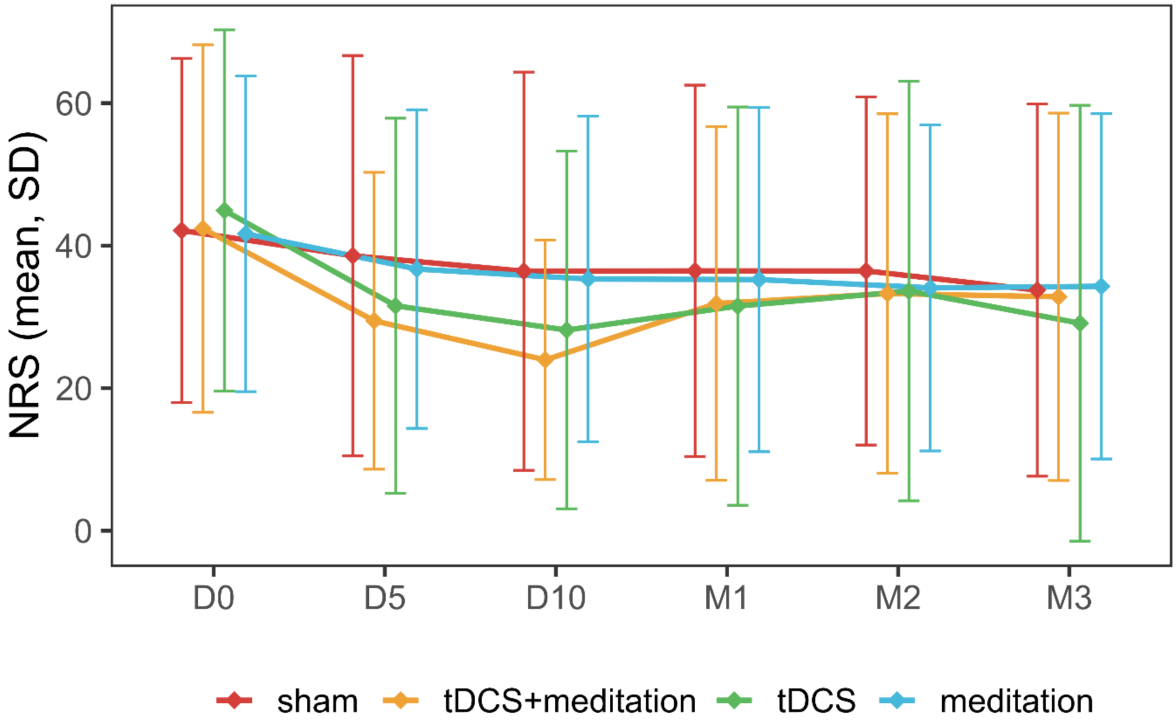

Section/topic	No	CONSORT 2025 checklist item description	Reporte[illegible] on page [illegible]
**Title and abstract**			
Title and structured abstract	1a	Identification as a randomised trial	1
	1b	Structured summary of the trial design, methods, results, and conclusions	2
**Open science**			
Trial registration	2	Name of trial registry, identifying number (with URL) and date of registration	14
Protocol and statistical analysis plan	3	Where the trial protocol and statistical analysis plan can be accessed	14
Data sharing	4	Where and how the individual de-identified participant data (including data dictionary), statistical code and any other materials can be accessed	19
Funding and conflicts of interest	5a	Sources of funding and other support (eg, supply of drugs), and role of funders in the design, conduct, analysis and reporting of the trial	19
	5b	Financial and other conflicts of interest of the manuscript authors	19
**Introduction**			
Background and rationale	6	Scientific background and rationale	3-4
Objectives	7	Specific objectives related to benefits and harms	4-5
**Methods**			
Patient and public involvement	8	Details of patient or public involvement in the design, conduct and reporting of the trial	n/a
Trial design	9	Description of trial design including type of trial (eg, parallel group, crossover), allocation ratio, and framework (eg, superiority, equivalence, non-inferiority, exploratory)	14
Changes to trial protocol	10	Important changes to the trial after it commenced including any outcomes or analyses that were not prespecified, with reason	n/a
Trial setting	11	Settings (eg, community, hospital) and locations (eg, countries, sites) where the trial was conducted	15
Eligibility criteria	12a	Eligibility criteria for participants	14-15
	12b	If applicable, eligibility criteria for sites and for individuals delivering the interventions (eg, surgeons, physiotherapists)	n/a
Intervention and comparator	13	Intervention and comparator with sufficient details to allow replication. If relevant, where additional materials describing the intervention and comparator (eg, intervention manual) can be accessed	15-16
Outcomes	14	Prespecified primary and secondary outcomes, including the specific measurement variable (eg, systolic blood pressure), analysis metric (eg, change from baseline, final value, time to event), method of aggregation (eg, median, proportion), and time point for each outcome	17-18
Harms	15	How harms were defined and assessed (eg, systematically, non-systematically)	5, 16
Sample size	16a	How sample size was determined, including all assumptions supporting the sample size calculation	18
	16b	Explanation of any interim analyses and stopping guidelines	n/a
Randomisation:			
Sequence generation	17a	Who generated the random allocation sequence and the method used	14
	17b	Type of randomisation and details of any restriction (eg, stratification, blocking and block size)	n/a
			**Reported o[illegible] page no.**

Allocation concealment mechanism	18	Mechanism used to implement the random allocation sequence (eg, central computer/telephone; sequentially numbered, opaque, sealed containers), describing any steps to conceal the sequence until interventions were assigned1	14
Implementation	19	Whether the personnel who enrolled and those who assigned participants to the interventions had access to the random allocation sequence	14
Blinding	20a	Who was blinded after assignment to interventions (eg, participants, care providers, outcome assessors, data analysts)	17
	20b	If blinded, how blinding was achieved and description of the similarity of interventions	16
Statistical methods	21a	Statistical methods used to compare groups for primary and secondary outcomes, including harms	18
	21b	Definition of who is included in each analysis (eg, all randomised participants), and in which group	18
	21c	How missing data were handled in the analysis	19
	21d	Methods for any additional analyses (eg, subgroup and sensitivity analyses), distinguishing prespecified from post hoc	18
**Results**			
Participant flow, including flow diagram	22a	For each group, the numbers of participants who were randomly assigned, received intended intervention, and were analysed for the primary outcome	Figure 1.
	22b	For each group, losses and exclusions after randomisation, together with reasons	Figure 1.
Recruitment	23a	Dates defining the periods of recruitment and follow-up for outcomes of benefits and harms	17-18
	23b	If relevant, why the trial ended or was stopped	n/a
Intervention and comparator delivery	24a	Intervention and comparator as they were actually administered (eg, where appropriate, who delivered the intervention/comparator, how participants adhered, whether they were delivered as intended (fidelity))	15-16
	24b	Concomitant care received during the trial for each group	n/a
Baseline data	25	A table showing baseline demographic and clinical characteristics for each group	Table 1
Numbers analysed, outcomes and estimation	26	For each primary and secondary outcome, by group:<br>● the number of participants included in the analysis<br>● the number of participants with available data at the outcome time point<br>● result for each group, and the estimated effect size and its precision (such as 95% confidence interval)<br>● for binary outcomes, presentation of both absolute and relative effect size	Figure 1 & Table 1
Harms	27	All harms or unintended events in each group	5
Ancillary analyses	28	Any other analyses performed, including subgroup and sensitivity analyses, distinguishing pre-specified from post hoc	18
**Discussion**			
Interpretation	29	Interpretation consistent with results, balancing benefits and harms, and considering other relevant evidence	9-13
Limitations	30	Trial limitations, addressing sources of potential bias, imprecision, generalisability, and, if relevant, multiplicity of analyses	12-13

Citation: Hopewell S, Chan AW, Collins GS, Hróbjartsson A, Moher D, Schulz KF, et al. CONSORT 2025 Statement: updated guideline for reporting randomised trials. BMJ. 2025; 388:e081123.
https://dx.doi.org/10.1136/bmj-2024-081123
© 2025 Hopewell et al. This is an Open Access article distributed under the terms of the Creative Commons Attribution License (https://creativecommons.org/licenses/by/4.0/), which permits unrestricted use, distribution, and reproduction in any medium, provided the original work is properly cited.

*We strongly recommend reading this statement in conjunction with the CONSORT 2025 Explanation and Elaboration and/or the CONSORT 2025 Expanded Checklist for important clarifications on all the ite
We also recommend reading relevant CONSORT extensions. See www.consort-spirit.org.
